# Texture and sensory characterization of functional yogurt supplemented with flaxseed during cold storage

**DOI:** 10.1002/fsn3.805

**Published:** 2019-02-13

**Authors:** Malihe Mousavi, Ali Heshmati, Amir Daraei Garmakhany, Aliasghar Vahidinia, Mehdi Taheri

**Affiliations:** ^1^ Department of Nutrition and Food Hygiene School of Medicine Nutrition Health Research Center Hamadan University of Medical Sciences Hamadan Iran; ^2^ Department of Food Science and Technology Toyserkan Faculty of Industrial Engineering Bu‐Ali Sina University Toyserkan City, Hamedan Iran

**Keywords:** flaxseed, functional food, sensory characterization, texture analysis, yogurt

## Abstract

In this study, flaxseed was used as a functional ingredient in yogurt formulations. The goal of this study was to produce prebiotic yogurt supplemented with flaxseed and investigation of its texture and sensory properties. Yogurt samples containing 0%–4% flaxseed was produced and stored at refrigerator (4–5°C) for 28 days. Textural properties were determined by texture analysis, and sensory characteristics were assessed by 26 trained panelists. Addition of flaxseed to yogurt samples increased the hardness, gumminess, chewiness, cohesiveness, and springiness values in produced yogurt samples. However, adhesiveness level was reduced in a sample enriched with flaxseed. By increasing flaxseed concentration, the color of samples was significantly different than the control sample; L* value was diminished and a* and b* value increased. Sensory scores including taste and mouthfeel, appearance, and overall acceptance showed reduction trend in samples containing a high level of flaxseed. In general, results showed that the addition of 2.63% flaxseed into yogurt samples lead to produce functional food with satisfactory texture, sensory characteristics that sustained these properties until 17.17 days after cold storage.

## 1 INTRODUCTION

In recent years, the knowledge of consumers about food ingredients and their associated health benefits has been considerably increased (Brouns & Vermeer, 2000). Therefore, there is a requirement for foodstuff production with health benefits and people are making a conscious attempt to include them in their diet, with the hopes of maintaining or increasing their life quality (Ahmad, Yap, Kofli, & Ghazali, 2018; Brouns & Vermeer, 2000). Functional foods are expressed as “foods that through specific beneficial physiological action, improve the health of the consumer” (Corbo, Albenzio, De Angelis, Sevi, & Gobbetti, 2001). Today, nutritionists believed that dairy products are beneficial for human health, due to high digestibility and nutritional value (García‐Pérez et al., 2005; Sadeghi, 2016). Also, studies were done to incorporate fiber into dairy products to increase their health improving properties. In this regard, the addition of plant sources materials that contain significant amounts of fiber (such as adding rice bran, wheat fiber, inulin, etc.) to dairy products has been considered (Hasani, Khodadadi, & Heshmati, 2016; Heshmati, Hasani, & Sari, 2016). Yogurt is the most popular fermented dairy product generally combined with fruits and fibers (García‐Pérez et al., 2005). Although dairy products are said to have high nutritional value, there is a lot of controversy about the health of their fats. Most scientific sources emphasize that fatty dairy products are not suitable for humans due to trans fatty acids, cholesterol, and saturated fatty acids. So much emphasis is placed on replacing fats of dairy products with appropriate vegetable fats (Bermúdez‐Aguirre & Barbosa‐Cánovas, 2011).

Flaxseed is one of the best sources of omega‐3 fatty acids or alpha‐linolenic acid, generally constituting 50%–62% of the total fatty acids of this seed (Daun, Barthet, Chornick, & Duguid, 2003). It is one of the good sources of fiber (10%) that could be introduced as a functional food (Oomah, 2001; Rubilar, Gutiérrez, Verdugo, Shene, & Sineiro, 2010). Therefore, the addition of flaxseed into yogurt samples will make consumers more interested to use this nutrient seed.

Textural and sensory properties are two important factors for assessment of Yogurt quality. Adding flaxseed into Yogurt samples can affect color, texture, and sensory properties of the final product. Hardness, adhesiveness, cohesiveness, gumminess, chewiness, and springiness properties are the most important parameters for the textural assessment of yogurt and other fermented milk products (Magenis et al., 2006). Therefore, it is necessary to find optimum flaxseed concentration that could be added into the yogurt samples to manufacture a product with excellent texture, sensory, and color attributes that sustained these properties during certain storage time.

## 2 MATERIALS AND METHODS

### 2.1 Materials

Low‐fat milk (1.5%) obtained from Pegah company (Hamadan, Iran) and starter culture containing *Streptococcus thermophilus* and *Lactobacillus delbrueckii* subsp. *bulgaricus* was bought from Chr. Hansen (Copenhagen, Denmark). Flaxseed was purchased from the local market (Hamadan, Iran) and powdered and sieved before incorporation into the yogurt samples. Other chemicals were from Merck Company (Darmstadt, Germany).

### 2.2 Yogurt samples preparation

Two types of yogurt samples were produced: control yogurt sample (without flaxseed) and prebiotic yogurt samples (containing 2% and 4% flaxseed). For preparing control yogurt, homogenized milk was heated at 90°C for 10 min then cooled to 43°C in an ice water bath and then were inoculated with starter cultures consisting of *S. thermophilus* and *L. delbrueckii* subsp. *Bulgaricus*. For prebiotic yogurt samples production, flaxseed was added to the raw milk. Then, the inoculated mix was incubated at 42°C to a final pH of 4.5 (5–6 hr). In final, samples were stored in the refrigerator for 28 days.

### 2.3 Textural profile analysis of yogurt samples

Textural parameters were measured by using texture profile analysis (TPA) (Zwick Company, Ulm, Germany) with mechanical compression of samples and the back extrusion test in four cycles with the cylindrically shaped probe (diameter of 40 mm). TPA instrument measured different parameters such as hardness, chewiness, gumminess, springiness, cohesiveness, and adhesiveness. The analyzer was connected to a computer that documented data via a software program called test software testXpert^®^ II.

### 2.4 Sensory evaluation

Five‐point hedonic scale test (including 1 = dislike very much; 2 = “dislike”, 3 = “neither like nor dislike”, 4 = like and 5 = like very much) was used for evaluating sample acceptability. Sensory assessment of yogurt samples was done after 1, 14, and 28 days of cold storage. Twenty‐six persons as panelists (that were members of the staff and students of the Hamedan University of medical science, Iran) assessed the sensory properties of yogurt samples. For this experiment, the cups that contained 100 ml of yogurt sample at 10°C were provided. Each sample was assessed by a person with three repeats. Yogurt samples were assessed for flavor, mouth feels, appearance, non‐mouth feel properties, and overall acceptability.

### 2.5 Color evaluation

Color measurement was done similar to the previous study with some modifications (Khodadadi, Ardebili, Eyvazzadeh, Zargari, & Moradi, 2014). Yogurt samples were placed in the floor of the aluminum dark chamber with 30 × 40 × 40 cm dimensions. A digital camera (Canon, Japan) was located on the roof of the chamber, and four 60‐w halogen lamps were placed in chamber inner corners. The samples were placed on the floor of the chamber. The images captured by mentioned camera were transferred to a computer, and its color was measured by image processing software (Photoshop CS 5 Portable) according to the Hunter Lab format that is L* (brightness), a* (+ red to –green component) and b* (+yellow to – blue component). Also, the color change was calculated as ∆E according to the following equation:

$$\Delta E=\sqrt{\Delta L^{*2}+\Delta a^{*2}+\Delta b^{*2}}$$

### 2.6 Experimental designs and analysis of data

In this study, response surface method (RSM) was used to determine the effect of independent variables on textural and sensory properties of produced yogurt samples. Independent variables were storage time (X1), flaxseed concentration (X2) and responses were hardness (Y1), adhesiveness (Y2), cohesiveness (Y3) gumminess (Y4), springiness (Y5), chewiness (Y6), taste (Y7), mouthfeel (Y8), appearance (Y9), overall acceptability (Y10), L* value (Y11), b* value (Y12), a*value (Y13), ∆E (Y14). Obtained design from design expert software contained 13 runs. The range of variables chosen for flaxseed concentration and storage time was 2%–4% and 1–28 days, respectively.

Software (Design‐Expert 7.0.0) was applied for statistical data analysis. Model, lack‐of‐fit, pure error, and other statistical results were calculated and showed in (Table 1).

**Table 1:** ANOVA for responses by response surface method

Response	Source	Sum of squares	*DF*	Mean square	*F* value	Prob > *F*	Model
Hardness (N), Y1	Model	697.95	5	139.59	196.62**	<0.0001	Quadratic
	Lack of fit	0.46	3	0.15	0.14	0.9339	
	Pure Error	4.51	4	1.13			
	Total	702.92	12				
Adhesiveness (N), Y2	Model	241.95	5	48.39	647.63**	<0.0001	Quadratic
	Lack of fit	0.21	3	0.070	0.89	0.5184	
	Pure Error	0.31	4	0.078			
	Total	242.47	12				
Cohesiveness (N), Y3	Model	5.052E‐003	5	1.010E‐003	91.80**	<0.0001	Quadratic
	Lack of fit	2.456E‐005	3	8.185E‐006	0.62	0.6362	
	Pure Error	5.249E‐005	4	1.312E‐005			
	Total	5.129E‐003	12				
Gumminess (N), Y4	Model	1.72	5	0.34	43.90**	<0.0001	Quadratic
	Lack of fit	0.041	3	0.014	4.12	0.1026	
	Pure Error	0.013	4	3.354E‐003			
	Total	1.77	12				
Springiness (N), Y5	Model	0.035	3	6.971E‐003	53.91**	<0.0001	Quadratic
	Lack of fit	1.886E‐004	3	6.286E‐005	0.35	0.7920	
	Pure Error	7.166E‐004	4	1.792E‐004			
	Total	0.036	12				
Chewiness (N), Y6	Model	0.11	2	0.057	371.41**	<0.0001	Linear
	Lack of fit	1.039E‐003	6	1.732E‐004	1.42	0.3827	
	Pure Error	4.877E‐004	4	1.219E‐004			
	Total	0.11	12				
Taste (score), Y7	Model	4.60	5	0.92	6.23*	0.0163	Quadratic
	Lack of fit	0.65	3	0.22	2.22	0.2284	
	Pure Error	0.39	4	0.097			
	Total	1.77	12				
Mouth feel, (score), Y8	Model	6.79	3	2.26	53.28**	<0.0001	2FI
	Lack of fit	0.18	5	0.036	0.73	0.6376	
	Pure Error	0.20	4	0.050			
	Total	7.17	12				
Appearance, (score), Y9	Model	0.75	3	0.25	3.62	0.0580	2FI
	Lack of fit	0.27	5	0.053	0.61	0.7036	
	Pure Error	0.35	4	0.088			
	Total	1.37	12				
Overall acceptability (score),Y10	Model	1.24	3	0.41	7.03**	0.0098	2FI
	Lack of fit	0.41	5	0.083	2.85	0.1657	
	Pure Error	0.13	4	0.029			
	Total	1.77	12				
L* value, Y11	Model	228.92	5	45.78	6.366E+007**	<0.0001	Quadratic
	Lack of fit	0.000	3	0.000			
	Pure Error	0.000	4	0.000			
	Total	228.92	12				
b* value,Y12	Model	14.31	5	2.68	6.366E+007**	<0.0001	Quadratic
	Lack of fit	0.000	3	0.000			
	Pure Error	0.000	4	0.000			
	Total	14.31	12				
a*value, Y13	Model	2.31	5	0.46	6.366E+007**	<0.0001	Quadratic
	Lack of fit	0.000	3	0.000			
	Pure Error	0.000	4	0.000			
	Total	2.31	12				
Delta E, Y14	Model	242.55	5	48.51	6.366E+007**	<0.0001	Quadratic
	Lack of fit	0.000	3	0.000			
	Pure error	0.000	4	0.000			
	Total	242.55	12				

*Notes*

*DF*: degree of freedom.

*Significant at *p *<* *0.05; **Significant at *p *<* *0.01.

### 2.7 Optimization

For optimization, responses such as hardness, chewiness, springiness, cohesiveness, and independent variable including flaxseed concentration and storage time were selected in maximum and adhesiveness and gumminess in minimum. Another variable was selected according to Table 2.

**Table 2:** Optimization of independent variables and responses for flaxseed‐enriched yoghurt production

Constraints	Goal	Lower limit	Upper limit	Lower weight	Upper weight	Importance
X1:Storage time (day)	Maximize	1	28	1	1	3
X2:Flaxseed concentration (%w/w)	Maximize	0	4	1	1	3
Overall acceptability (score)	Maximize	3.6	5	1	1	3
Hardness (N)	Maximize	20.45	43.05	1	1	3
Adhesiveness (N)	Minimize	18.12	31.26	1	1	3
Springiness (N)	Maximize	0.7815	0.9323	1	1	3
Gumminess (N)	Minimize	0.4384	1.7365	1	1	3
Chewiness (N)	Maximize	0.5213	0.8011	1	1	3
Cohesiveness (N)	Maximize	0.6109	0.6815	1	1	3
Taste (score)	Maximize	2.1	5	1	1	3
Mouth feel (score)	Maximize	2.1	5	1	1	3
Appearance (score)	Maximize	3.8	5	1	1	3
Overall acceptance	Maximize	3.6	5	1	1	3
Color						
L* value	Maximize	56	68	1	1	3
b* value	In range	4	7	1	1	3
a* value	In range	−1	0	1	1	3
Delta E	In range	0	12.4	1	1	3

## 3 RESULTS AND DISCUSSION

### 3.1 Evaluation of hardness

**Figure 1:**
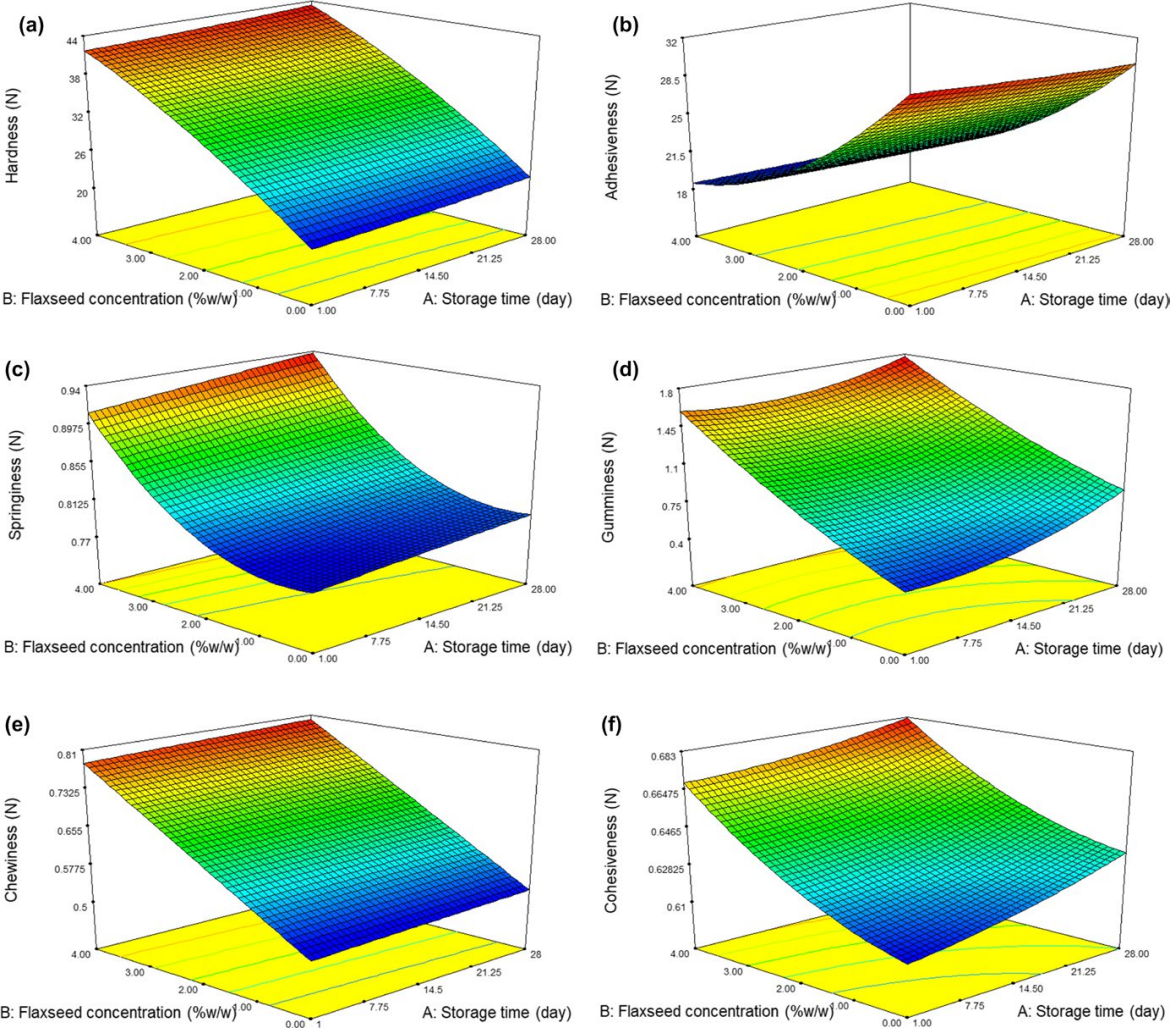
The effect of flaxseed concentration and storage time on Hardness (a), Adhesiveness (b), Springiness(c), Gumminess (d), Chewiness (e), and Cohesiveness (f) of flaxseed‐enriched yogurt samples

Hardness or firmness is the most commonly assessed parameter for yogurt texture analyses that it was defined as the necessary force to attain a given deformation. This factor is a critical texture property for yogurt like products. The findings obtained from ANOVA are shown in Table 1. The *F* and *p*‐values indicated that the quadratic model is suitable for hardness determination and was statistically significant at a 99% confidence interval. Also, the lack of a fit test for this variable is insignificant. Flaxseed caused a significant increase in hardness of the samples (*p *<* *0.05) while storage time had no considerable influence on this parameter (Table 1). The lowest (20.45 N) and highest amount of hardness (43.05 N) were found in control and yogurt sample containing 4% flaxseed, respectively (Figure 1). The higher hardness in yogurts fortified with flaxseed can be due to the presence of fiber in flaxseed. Fiber improves the growth of starter cutler of yogurt, that is, *L. delbrueckii* ssp. *bulgaricus* and *S. thermophilus*. When these bacteria grow well, could result in create desirable texture properties and consequently lead to an increase in yogurt hardness. Besides, hardness increment may be related to the moisture absorption ability of flaxseed. The amount of yogurt hardness was dependent on incorporated compound contents, starter culture level, and incubation time. Starter culture level could increase hardness in yogurt sample. However incubation time did not result in significant change in hardness (Mudgil, Barak, & Khatkar, 2017).

In this study, we found that flaxseed increased the yogurt hardness while some studies have been shown that the addition of functional compounds into yogurt lead to a decrease in hardness. Azari‐Anpar, Tehrani, Aghajani, and Khomeiri (2017), Azari‐Anpar, Payeinmahali, Daraei Garmakhany, and Sadeghi Mahounak (2017) showed that the addition of *Aloe Vera* gel into yogurt decreased samples harness so that the lowest of hardness was related to the sample containing 5% *Aloe Vera* gel. The occurrence of salicylic acid and antimicrobial agents in Aloe Vera gel decreased starter culture bacteria growth (Azari‐Anpar, Tehrani, et al., 2017). Also, Michael, Phebus, and Schmidt (2010) found that plant extracts including olive, onion, citrus, and garlic decrease the yogurt hardness or firmness (Michael et al., 2010). In another study, it was observed that the addition of 2‐2.5% partially hydrolyzed guar gum (PHGG) to yogurt samples did not influence on hardness while values greater than 2.5% reduced hardness (Mudgil et al., 2017).

### 3.2 Evaluation of adhesiveness

Adhesiveness or stickiness is the required work for prevailing attraction force between foodstuff surface and various substances coming into contact with them. In fact, adhesiveness is the force required to separate the material that sticks to the teeth during eating (Delikanli & Ozcan, 2017). Adhesiveness had an inverse relationship with yogurt eating quality. As can be seen from the ANOVA data in Table 1, the quadratic model obtained for adhesiveness was significant (*p* < 0.01) and lack of fit was not significant. In the current study, the highest amount of adhesiveness (31.26 N) was related to control yogurt while flaxseed‐enriched (4w/w) yogurt had the lowest (18.12 N) amount of adhesiveness (Table 2). The increment of storage time and flaxseed concentration resulted in adhesiveness reduction. Our finding was similar to the study by Azari‐Anpar, Tehrani, et al. (2017); Azari‐Anpar, Payeinmahali, et al. (2017) that found *Aloe Vera* foliar gel addition to yogurt samples reduced the adhesiveness of produced samples (Azari‐Anpar, Tehrani, et al., 2017). Also, Grega, Sady, Wszolek, and Gambus (2001) reported that the addition of amaranthus seeds into yogurt samples decrease the adhesiveness amount (Grega et al., 2001). In another study, it was revealed the replacement of milk fat with maltodextrin resulted in the increment of yogurt adhesiveness (Domagała, Sady, Grega, & Bonczar, 2006). In this study, we found that the adhesiveness decreased by increasing the storage time while the opposite results have been reported by some authors. Akgun, Yazici, and Gulec (2016) found that storage time has no significant (*p *>* *0.05) impact on the amount of adhesiveness in buffalo milk yogurt (Akgun et al., 2016). It seemed that flaxseed caused the formation of a weak three‐dimensional network in yogurt (Tavakolipour, Vahid‐moghadam, & Jamdar, 2014).

### 3.3 Evaluation of cohesiveness

Cohesiveness or consistency is an important textural parameter of yogurt and shows its acceptance from the consumer's point of view. Cohesiveness is defined as the forces of inner bond links, which maintain the product as a perfect, and it is expressed as the force content that can cause to deform a material before it is broken. As can be seen from Figure 1 and Table 1, flaxseed concentration and storage time had a significant impact on the samples cohesiveness (*p *<* *0.01). Flaxseed incorporation (4%) into the yogurt samples increased cohesiveness value from 0.61 (control sample) to 0.68 N. Since cohesiveness indicates the strength of the internal bonds in yogurt structure, therefore high value of cohesiveness showed that yogurt structure in samples containing flaxseed is more strength and firmer compared to control sample (Salvador & Fiszman, 2004). The protein matrix had an important role in cohesiveness (Tunick, 2000). Our result is opposite to the findings obtained for yogurt containing *Aloe Vera* foliar gel that decrease in cohesiveness reported in the final product (Azari‐Anpar, Tehrani, et al., 2017; Azari‐Anpar, Payeinmahali, et al., 2017). Also, it was reported that the addition of 3.0%, 4.0%, and 5.0% of dried grape pomace into the yogurt samples reduced cohesiveness amounts (Mohamed, Zayan, & Shahein, 2014). Domagała et al. (2006), showed that there is no significant difference between cohesiveness of control yogurt and yogurts containing fat or maltodextrin (Domagała et al., 2006). The increment in cohesiveness during cold storage was reported in another study that confirms our finding. do Espírito Santo, Perego, Converti, and Oliveira (2012) found that the cohesiveness amount of yogurt containing passion fruit peel powder, increased during storage time (do Espírito Santo et al., 2012). Flaxseed influenced on internal bonds in yogurt structure, thus it reduced adhesiveness and increased cohesiveness (Bhat, Deva, & Amin, 2018).

### 3.4 Evaluation of gumminess

Gumminess is defined as the energy required to break a semisolid food into fragments until it is ready to swallow (Dar & Light, 2014; Domagała et al., 2006;). It is a defect. The range of gumminess was 0.43 and 1.73 N (Table 2). The lowest and highest gumminess value was related to control (0.43 N) yogurt and yogurt containing 4% flaxseed (1.73 N), respectively. The results showed that the quadratic model for flaxseed concentration and storage time were significant (*p *<* *0.01). While lack‐of‐fit for gumminess was insignificant value. Gumminess has an undesirable effect on appearance and texture. Hence, high flaxseed level had a negative effect on gumminess, and since the flaxseed had an intense effect on texture, so we observed the increment of gumminess for flaxseed‐enriched yogurt. Our result was similar to Azari‐Anpar, Tehrani, et al. (2017); Azari‐Anpar, Payeinmahali, et al. (2017) that found the addition of gums caused enhancement in gumminess. These authors found there is an effective relationship between the fermentation velocity and gumminess (Azari‐Anpar, Tehrani, et al., 2017; Azari‐Anpar, Payeinmahali, et al., 2017). Besides, in other research, it was found that incorporation of amaranthus ground seeds into yogurt, enhanced gumminess (Domagała et al., 2006). However, there are opposite results with our results. So that, Nikoofar, Hojjatoleslami, and Shariaty (2013) observed that fortification of yogurt with Quince seed mucilage decreased gumminess of the final product (Nikoofar et al., 2013).

### 3.5 Evaluation of springiness

Springiness is the rate and extent to which a deformed material returns to its initial condition after the force is eliminated. Springiness depends on different agents such as heat treatment, protein interaction, elasticity, and degree of unfolding of protein (Delikanli & Ozcan, 2017). The influence of flaxseed addition to yogurt on textural profile properties of springiness is presented in (Table 2). There were significant differences in the textural properties of springiness between flaxseed‐enriched and control samples (*p *<* *0.05). But the effect of flaxseed concentration levels on the springiness of yogurt samples was more important than the effect of storage time. The springiness of yogurt samples was reported maximum at levels 4% of flaxseed and storage time of 28 days. The amount of springiness was between 0.78 and 0.93N (Table 2). Springiness indicates the texture integrity of yogurt and addition of flaxseed in yogurt, increase texture integrity, so this is a suitable reason for the higher springiness observed in yogurt containing flaxseed compared to control sample. Mudgil et al. (2017) Studies were in agreement with our result; they showed that springiness of the yogurt samples increased by the increase in (partially hydrolyzed guar gum) PHGG level (Mudgil et al., 2017). Ayar and Gurlin (2014) found that springiness has enhanced in the yogurt samples fortified with carrot during 1st and 10th days of storage time (Ayar & Gurlin, 2014). However, there are opposite studies with our studies, for instance, Mudgil et al. (2017) showed that springiness of yogurt samples decreased with an increase in incubation time (Mudgil et al., 2017).

### 3.6 Evaluation of chewiness

The chewiness is the time or work needed for masticating a sample for decrease it to a state ready for consuming; it is related to firmness, cohesiveness, and elasticity (Dar & Light, 2014). Response surface plots of chewiness for yogurt samples as a function of flaxseed concentration and storage time are shown in Figure 1. Model of RSM for chewiness was linear and significant. Chewiness of our samples ranged from 0.52 to 0.80 N (Table 2) depending on flaxseed levels and storage time. Amount of flaxseed has a significant effect on chewiness, while storage time did not affect this parameter. The increase in chewiness of the yogurt samples containing flaxseed could be due to the viscosity effect of flaxseed that might have further improved the structure of the yogurt sample. In a study by Azari‐Anpar, Tehrani, et al. (2017); Azari‐Anpar, Payeinmahali, et al. (2017), obtained findings were contrast with our studies. They indicated that the addition of *Aloe Vera* gel concentration to yogurt leads to a significant decrease in chewiness (Azari‐Anpar, Tehrani, et al., 2017; Azari‐Anpar, Payeinmahali, et al., 2017).

### 3.7 Sensory evaluation

**Figure 2:**
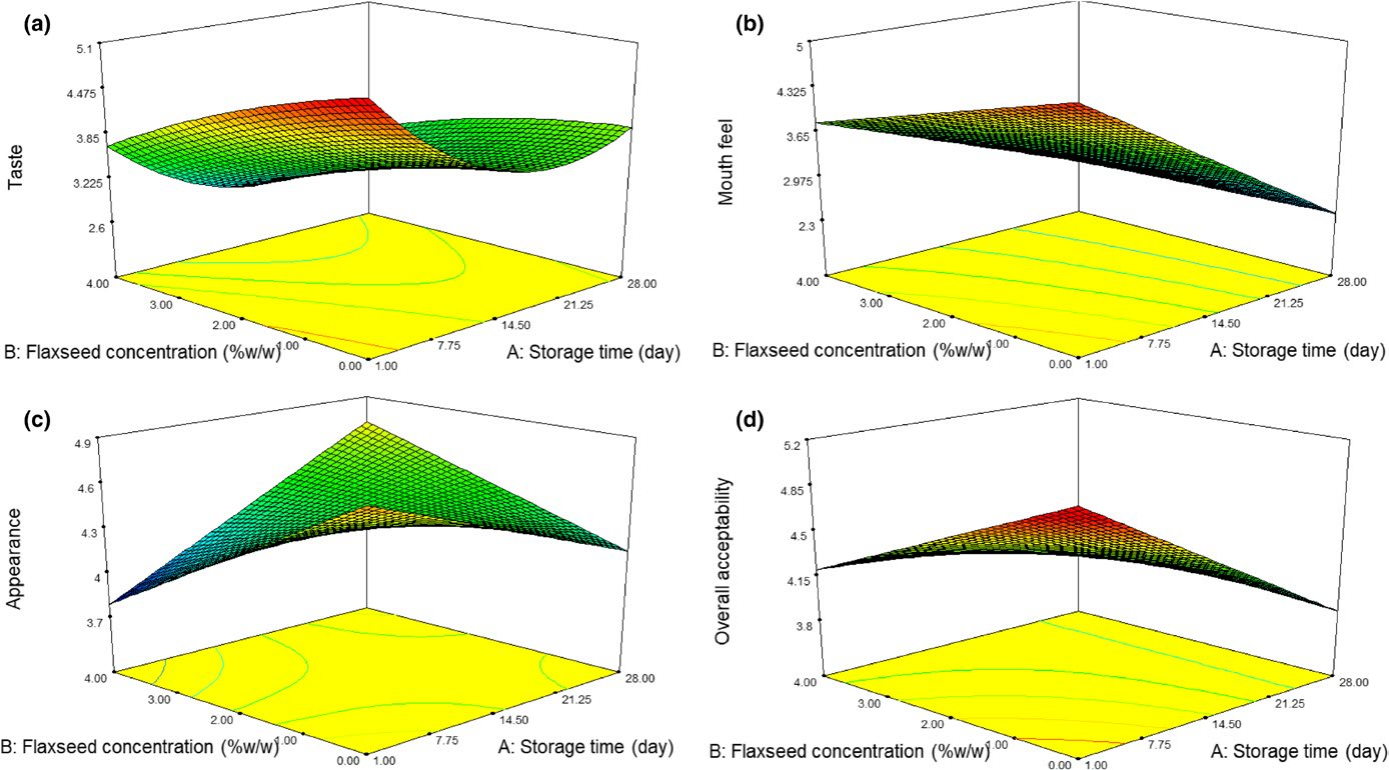
Response surface plot of the effects of flaxseed concentration and storage time on sensory properties including: taste (a), mouth feel (b), appearance (c) and overall acceptability (d) of flaxseedenriched yogurt samples

Sensory assessment helps to define the product properties which are prominent concerning the product acceptability for the customer. In this study, the influence of flaxseed concentration and storage time on the sensorial properties of the flaxseed‐enriched and control yogurt samples is represented by the response surface plots for better conception in Figure 2. Sensorial properties such as taste, flavor, appearance, mouthfeel, and overall acceptability were considered to evaluate the sensory quality of the final product. Flaxseed concentration and storage time affect the sensory properties of different types of yogurt samples significantly. RSM Model for the taste of this kinds of yogurt is quadratic (Table 1) and significant. The panelists gave the greater score of taste to the control yogurt in first day of storage time. Results from the mouthfeel score showed that panelists dislike this parameter in flaxseed‐enriched yogurt in comparison with control sample. The characteristics of the appearance of yogurt samples are shown in Figure 2. The appearance score was from 3.8 to 5 N. The overall acceptability of flaxseed‐enriched yogurt sample was significantly (*p *<* *0.01) reduced by increasing storage time and flaxseed concentration. By increase storage time and flaxseed concentration, the overall acceptability of yogurt samples was reduced.

The addition of dried grape pomace, rice, and bare bran into the yogurt samples reduced the appearance, Flavor, texture, and overall acceptability score compared with control yogurt (Hasani, Sari, Heshmati, & Karami, 2017; Hasani et al., 2016; Mohamed et al., 2014).

Although sensory attribute score of flaxseed enriched yogurt samples is lower than control sample, it seemed that consumers preferred functional food with potential health advantages due to their nutritional information increment (Hasani et al., 2016). As well as, the addition of a flavoring agent into flaxseed enriched yogurt could improve the sensory characteristic of these products.

### 3.8 Color

**Figure 3:**
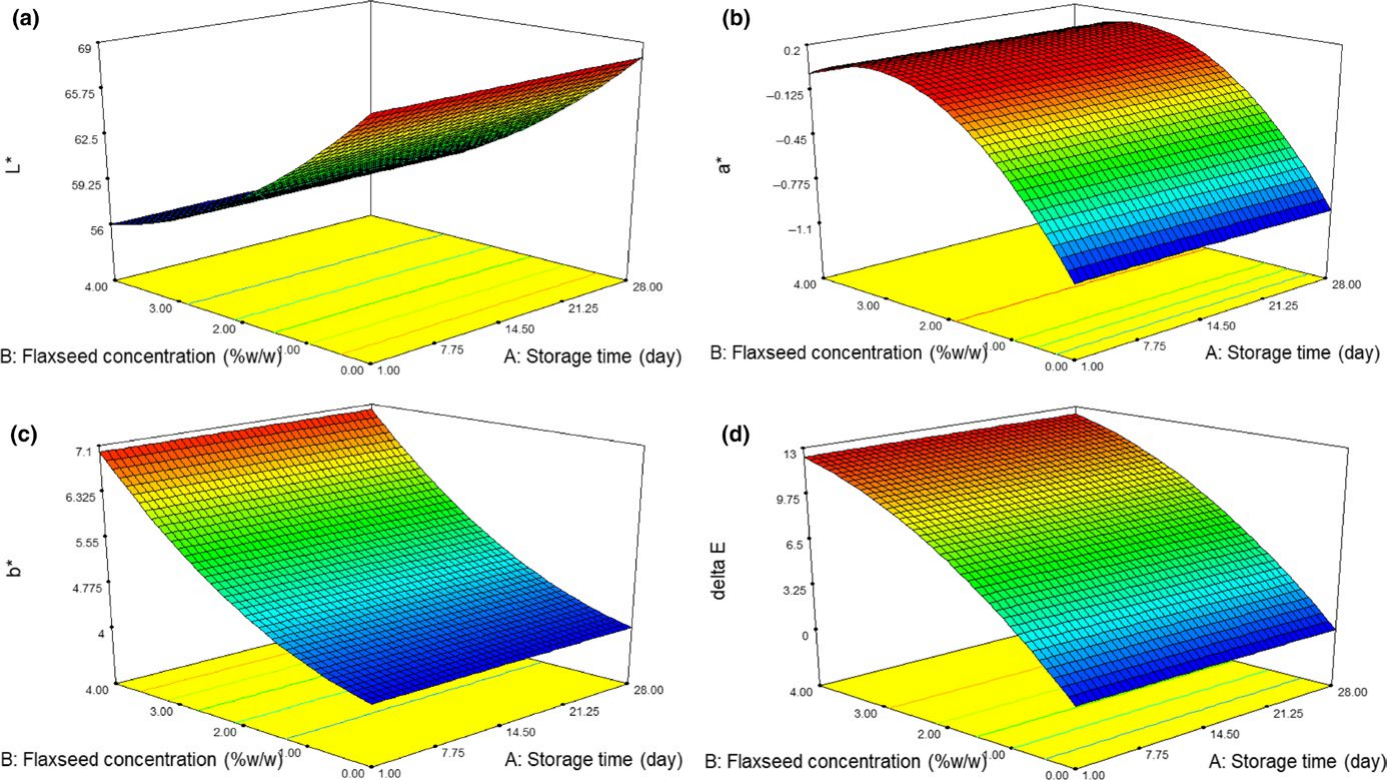
Response surface plot for color assessments. L* (a), a* (b), b* (c) and Delta E (d) in flaxseedenriched yogurt samples

The white color of milk, and also yogurt, is resulted in the light dispersion of fat globules and casein micelles (Walstra, Geurts, Walstra, & Wouters, 2005). In this study, we found that by addition of flaxseed, the yogurt color changed significantly (*p < *0.01). Although, storage time had no significant effect on the color intensity of yogurt samples (Figure 3). The L* value of the yogurt samples ranged from 56 to 68 (Table 2). The lightest (56) and the darkest (68) sample was observed in control and flaxseed‐ enriched (4%) yogurt, respectively. The flaxseed contains high fiber content and this component could absorb water and decrease the L* value of the yogurt samples; therefore, fiber displayed a darkening effect. Also, The results of b* values were significantly affected by flaxseed. So, the addition of flaxseed (due to fiber content) increased b* values. Pasteurization of milk containing flaxseed, released some of the pigments from flaxseed fiber, mainly flaxseed, making the product more yellow; even though it could not be seen by the human eye, it was detected by a colorimeter. Furthermore, pasteurization caused instability of the casein micelles that increases b* values. The a* values of flaxseed‐enriched yogurt samples at 28‐day of storage period were significantly higher than the control sample, that may be due to the red pigmentation of the flaxseed itself. Also ∆E was used to evaluate the overall color changes in the samples. Our result was similar to García‐Pérez et al. (2005) study which found that when orange fiber percentage was an enhancement in yogurt samples, an increase of a* (less greenness) and b* values (more yellowness) and a decrease in L* values (less whiteness) were reported (García‐Pérez et al., 2005).

Noh, Seo, Lee, and Chang (2013) found that the addition of Corni Fructus extract (CFE) into yogurt samples had no significant effect on the L*‐values. However, the a* and b* values significantly increased with the addition of CFE during storage period (Noh et al., 2013).

### 3.9 Optimization

**Figure 4:**
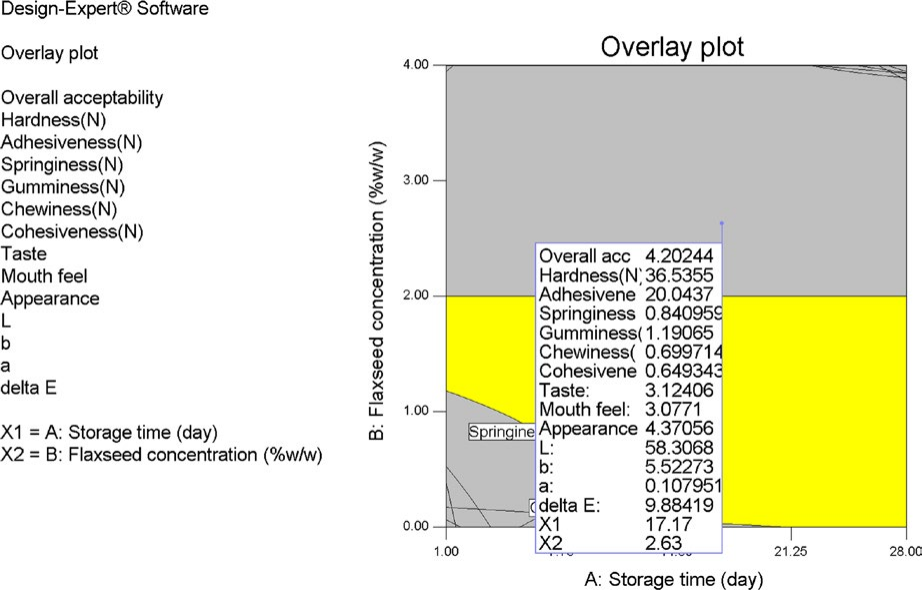
Overlay plot reporting the optimum levels product variables and responses values

The object of optimization is to obtain flaxseed‐enriched yogurt product with the good rheological, sensory, and color properties. So, the RSM was used in this study. According to the results, it can be said that flaxseed concentration and storage time as independent variables are the affecting factors on responses such as rheological properties, color index and sensory characteristics of yogurt samples. The condition applied to optimize flaxseed–enriched yogurt production is presented in Table 2. Overlay plot contour was used to select the best optimization condition (Figure 4) as well as to present suitable responses under this condition. In general, we found that incorporation of 2.63% flaxseed into yogurt samples lead to manufacturing a dairy product with acceptable texture, sensory, and color properties which is comparable to control yogurt sample until 17.17 days after a cold storage period.

## 4 CONCLUSION

Response surface method has been used for survey of the rheology, color indexes, and sensory properties of flaxseed‐enriched yogurt samples. Analysis of rheology characteristics is considered as a useful procedure for assessing the hardness, adhesiveness, cohesiveness, chewiness, gumminess, and springiness in kinds of yogurt. The results showed that the variables of the flaxseed concentration and storage time in flaxseed‐enriched yogurt samples resulted in the significant increment of all mentioned rheological parameters, except of adhesiveness. Whereas sensory properties were decreased in this product compared to control yogurt sample. In addition, L* value as color index diminished in samples containing flaxseed, although, a* and b* values in this product were greater than control yogurt sample. Generally, addition of 2.63% flaxseed into yogurt samples could produce functional food with satisfactory texture and sensory characteristics that sustained these properties until 17.17 days after cold storage. Flaxseed‐enriched yogurt samples with suitable quality in industrial scale can be achieved by use of optimization amounts obtained by RSM in this study. Therefore, our finding could be utilized for the production of flaxseed‐enriched yogurt as a functional food at commercial level.

## CONFLICT OF INTEREST

None declared.

## ETHICAL STATEMENT

Protocols and procedures in this study were ethically reviewed and approved by the Research Ethics Committee of Hamadan University of Medical Sciences, Hamadan, Iran.
